# Public engagement is key for the future of science research

**DOI:** 10.1038/npjscilearn.2016.10

**Published:** 2016-06-01

**Authors:** Daniel Pham

**Affiliations:** 1 Solomon Snyder Department of Neuroscience, Johns Hopkins University, Baltimore, MD, USA

I am a doctoral candidate in neuroscience, and I am planning to leave bench research behind following the completion of my degree. My goal is to advocate for science research through a career in science outreach and policy. Many support my decision and have expressed their excitement about my proposed trajectory. However, I have encountered some cooler reactions from academics, ranging from disappointment to betrayal. Given their success in navigating through their own careers, however, these responses make sense. Many faculty members have been successful without engaging the public. However, this strategy of isolation is not effective in today’s world. In the face of a rapidly changing society, science researchers must learn from Darwin and evolve.

The Pew Research Group and the American Association for the Advancement of Science published a recent survey that yielded interesting insights into how scientists think about public outreach.^1^ First, it illustrated the stark differences in the opinions between scientists and the general public. Asked for their opinions on whether humans have evolved over time, whether genetically modified foods are safe to eat and whether climate change is due to human activity, a 30-point difference or more was observed between scientists and the public. This discrepancy highlights the scientific community’s inability to effectively communicate our findings to the public. Another set of survey results illustrates a catch-22 in how scientists approach public outreach. A majority of scientists believe that the public is interested in their specialty area. They also contend that the media oversimplifies science, and that not enough is done to differentiate between well founded and poorly supported scientific ideas. However, the same survey shows that a majority of scientists believes that the promotion of one’s scientific findings to the public via the news or social media is irrelevant to their own career advancement. The results show a major breakdown of communication between scientists and the public. We recognise the miscommunication, yet we are not willing to address the issue because we believe it is not worth the effort and time.

Our lack of desire to communicate science to the public has created major problems for the science community. It is difficult to generate support from the public and the government if our audience does not understand the importance or relevance of our work. Moreover, doctoral students and postdoctoral researchers are not encouraged to use their training and expertise outside academia. Issues like a stagnant National Institutes of Health budget, the lack of diverse careers within and beyond academia and mistrust of science by the general public can be addressed if we all are involved in public engagement. Traditional approaches to science research have hampered our ability to respond to these issues. The following points below address these traditional and problematic perceptions I have heard directly from faculty about science research.

## Doctoral programs must focus on training new scientists to become academic professors

This line of thinking purports that any activities beyond the lab are inappropriate in a doctoral training program. Statistics inform us that not all PhD candidates are guaranteed an academic professorship. Only 15% of American graduate students ended up in tenured positions in 2006, compared with 55% in 1973.^2^ The lack of job opportunities has caused a rise in the number of postdocs unable to secure a faculty position. From 2000 to 2012, the number of postdocs increased by 150%, whereas the number of faculty openings decreased.^3^ Current doctoral programs must prepare students for diverse career options that allow them to utilise their skills and knowledge. Beyond the better-known paths in industry, as well as science writing, there is room for doctoral candidates to make their impact in fields beyond academia as policy analysts, consultants, entrepreneurs and innovators.

## As scientists, it is not our job to advocate for a larger federal budget

This is the common narrative of researchers being stuck in the labs, too busy to engage in advocacy work. However, performing science research in an isolated bubble is unsustainable. In the last decade, there has been a precipitous drop in basic science research funding.^4^ Instead of fighting for a shrinking slice of the pie, we must speak up to increase our share. Scientists are trained to be restrained and thorough, relying on the data to support our claims. However, this apprehensive thinking hurts our lobbying power. In 2006, the Federation of American Societies for Experimental Biology, boasting 27 scientific societies and >120,000 members, spent $160,000 on congressional lobbying efforts. In 2007, Research!America, a group focused on increasing funding for medical and health research, spent $140,000. In contrast, Focus on the Family, a Christian non-profit organisation that opposes stem cell research, spent $700,000 on lobbying efforts in 2005 and $3.9 million on public awareness efforts in 2007.^5^ This discrepancy in lobbying efforts has clear impacts on how society perceives science research. Furthermore, only 8.5% of PhDs in the United States are employed in government jobs.^6^ In countries like Austria and Spain, >20% of PhDs are in government jobs. The lack of scientists in government positions also hinders our ability to effectively advocate for our science. No one will suddenly realise the importance of our work and lobby on our behalf. Even if scientists cannot financially contribute to these lobbying efforts, we must realise the importance of these advocacy efforts and make them a priority.

## Science outreach is an ineffective way to further one’s science career

The lack of diverse jobs after a PhD, a shrinking funding budget and lack of public support all seem daunting to graduate students. In the face of these major hurdles, many scientists isolate themselves from these issues and focus on their own research. A recent study has shown, however, that increased interaction with reporters and the more Twitter mentions a study receives correlate with a higher *h*-index of the author, a metric for measuring the scientific impact of a publication.^7^ To determine whether media coverage of a published science article is causative of increased citations, a 1991 study looked at journal articles that would have been covered by the New York Times, but due to a writer’s strike in the late 1970s, were not. Researchers found that the journal articles that were not covered had consistently less citations than other research articles covered by the Times.^8^ There is clear value for the science research community to publicise new findings to the public.

## There is little scientific evidence of the efficacy of public engagement

Even though the societal impact of the science community’s public engagement has been difficult to measure,^9^ available studies show a general positive correlation between high-quality community engagement and positive public attitudes towards science research.^10^ A study surveying the impact of visiting the National Aquarium in Baltimore showed that changes in knowledge and interest in conservation persisted for 2 months after a single visit.^11^ A separate impact study that analysed over 180 evaluations of science centres around the world suggested that science-centre visits increased museum-goers’ interest in science.^12^ In addition, a report showing that increased exposure to science topics can lead to less support for biomedical research concedes that individuals exposed to high-quality science, such as that found in Scientific American, have a positive outlook towards this research.^13^ This emphasises that not only do we have to engage the community, but we must be properly trained and supported to ensure that our interactions are effective.

Science outreach is the first step in regaining the public’s trust. Many scientific societies, academic institutions and non-profit organisations have an outreach arm whose goal is to educate the public about scientific topics. The best way to practice public outreach is to begin with family and friends, who will gain a better appreciation for science and the people involved in the work. Public engagement, whether it is face-to-face or over social media, will encourage people to think actively about science and understand its relevance to them in their daily lives. Increased interaction with the public will effectively inspire new champions for science research and encourage them to lobby for science. This type of indirect grassroots lobbying has been effective in eliciting policy changes.^14^ Positive interaction between scientists and the public only builds a stronger coalition to eventually effect change.

The lack of diverse career options in academia, and also lack of faculty positions, stem from a decreasing budget unable to provide more jobs. As we engage in science outreach, we also change how the public views scientists. They will realise our skills and value. We need to further encourage scientists to consider other fields in which our knowledge and skill sets can be used to help others. This encouragement needs to come from the top. Today, Johns Hopkins University and other universities are trying to effect change by providing workshops on diverse opportunities in areas including biotech, law and policy. This is commendable, but scientists still have a long way to go. We must change our own hearts and minds before we can change the hearts and minds of others.
